# From the Clinical Mycology Laboratory: New Species and Changes in Fungal Taxonomy and Nomenclature

**DOI:** 10.3390/jof4040138

**Published:** 2018-12-16

**Authors:** Nathan P. Wiederhold, Connie F. C. Gibas

**Affiliations:** Fungus Testing Laboratory, Department of Pathology and Laboratory Medicine, University of Texas Health Science Center at San Antonio, San Antonio, TX 78229, USA; gibas@uthscsa.edu

**Keywords:** taxonomy, fungal nomenclature, phylogenetics, species complex

## Abstract

Fungal taxonomy is the branch of mycology by which we classify and group fungi based on similarities or differences. Historically, this was done by morphologic characteristics and other phenotypic traits. However, with the advent of the molecular age in mycology, phylogenetic analysis based on DNA sequences has replaced these classic means for grouping related species. This, along with the abandonment of the dual nomenclature system, has led to a marked increase in the number of new species and reclassification of known species. Although these evaluations and changes are necessary to move the field forward, there is concern among medical mycologists that the rapidity by which fungal nomenclature is changing could cause confusion in the clinical literature. Thus, there is a proposal to allow medical mycologists to adopt changes in taxonomy and nomenclature at a slower pace. In this review, changes in the taxonomy and nomenclature of medically relevant fungi will be discussed along with the impact this may have on clinicians and patient care. Specific examples of changes and current controversies will also be given.

## 1. Introduction

Kingdom Fungi is a large and diverse group of organisms for which our knowledge is rapidly expanding. This kingdom includes numerous species that are capable of causing disease in humans, animals and plants. Infections caused by fungi are highly prevalent in humans, as it is estimated that greater than 1 billion people worldwide have infections caused by these organisms [1,2]. However, the full extent of fungi capable of causing infections in humans remains unknown. Although only several hundred species have been reported to cause disease in humans [3], it is estimated that there are between 1.5 million to 5 million fungal species and only approximately 100,000 species have been identified [4,5]. The potential clinical relevance of yet to be discovered species is highlighted by the nearly 10-fold increase in reports of newly described fungal pathogens in plants, animals and humans since 1995 [6], as well as by outbreaks of infections caused by fungi previously not associated with severe disease in humans [7,8,9,10]. Those that are capable of causing systemic infections in humans often have key attributes that make this possible (e.g., growth at 37 °C, penetrate or circumvent host barriers, digest and absorb components of human tissue, withstand immune responses of host) [11]. Many are also capable of persisting in the environment due to saprobic potential (i.e., the ability to grow on dead or decaying material) [12]. In addition, many species may be generalist pathogens with little host specificity and have dynamic genomes allowing for rapid adaption and evolution [11,12,13]. Thus, the number of fungal species that are etiologic agents of human infections will continue to grow. As the number of pathogenic species continues to grow, many of which are opportunists, new classifications and nomenclature will be introduced. In addition, revisions to current taxonomy will continue to be made based on our increased understanding of the diversity of this kingdom. In this review, changes in taxonomy and nomenclature of clinically relevant fungi will be discussed as will the challenges posed to clinicians and clinical microbiology laboratories by these changes.

## 2. Changes in Fungal Taxonomy and Nomenclature

Over the last several years significant changes have occurred in fungal taxonomy and nomenclature, as new fungi are discovered and the relationships of individual species to others and within larger taxonomic groups have been re-evaluated and redefined. Although the discovery of new fungal species and their classification has been a continuous process since the advent of the field of mycology, the pace of discovery and re-evaluation of taxonomic status has increased with the introduction of molecular and proteomic tools. Historically, morphologic characteristics and other phenotypic traits (e.g., growth on different media at different temperature, biochemical analysis) have been used for both taxonomic evaluation and species identification in clinical settings. However, the phenotypic traits that are observed may vary under different conditions and are thus subjective. Errors in species identification may occur because of this. DNA sequence analysis is now considered the gold standard for fungal species identification and has been a driving force for the increased pace of the discovery of new species and changes in fungal taxonomy and nomenclature [14,15,16]. Phylogenetic analysis based on the sequences of multiple loci within fungal DNA is often used for taxonomic designation of new species and in the re-evaluation of previous classifications that had been based solely on phenotypic characteristics. An advantage of phylogenetic analysis for taxonomic purposes is that close relatives become grouped together regardless of differences in morphology and these relationships may be useful for predicting pathogenicity and susceptibility to antifungal drugs [17]. These methods have led to the discovery of numerous cryptic species, which are indistinguishable from closely related species based on morphologic characteristics but can be identified by molecular means [18]. However, the use of phylogenetic analysis for taxonomic re-evaluation is not without its flaws, as the relationships created may be subject to change with increased understanding of fungal diversity since phylogenetic trees are highly subject to sampling effects [17]. In addition, no delimitation criteria exist above the species level [19]. Newer technologies, such as matrix assisted laser desorption ionization time-of-flight mass spectrometry (MALDI-TOF MS), are also being used with increased frequency for rapid species identification in clinical settings as well as for the taxonomic evaluation of fungi [20,21,22,23]. It should be noted that clinical laboratories may need to exercise caution in the adoption of these technologies for the identification of all fungal isolates until appropriately validated in the literature. Some examples of new and clinically relevant fungal species are listed in Table 1. Clearly, the description and recognition of new species helps to advance the field of medical mycology by increasing our understanding of the epidemiology of various fungal infections, the geographic distribution of species that cause these infections and how infections caused by different species may respond differently to treatment [24,25,26,27,28].

In addition to new tools for fungal identification and taxonomic re-evaluation, changes in fungal nomenclature have also been brought about by the elimination of the dual nomenclature system. When fungal taxonomy was based solely on morphologic characteristics, many fungi were forced to have multiple names describing either their sexual (teleomorph) or asexual (anamorph) life cycle stages under Article 59 of the International Code for Botanical Nomenclature. However, this dual nomenclature system became obsolete with the introduction of molecular tools since different morphologic stages are identical at the genetic level [17,19,29]. Thus, the system was abolished under the newly named International Code of Nomenclature of algae, fungi and plants in which fungi are now only to have one name [30]. However, decisions regarding which names to use have not always been straightforward. Some examples of clinically relevant changes in nomenclature for yeasts and molds are shown in Table 2.

## 3. Implications of Changes in Nomenclature for Medical Mycology

The abolishment of the dual nomenclature system and the introduction of molecular tools for species identification have implications for medical mycology. There is concern that these changes may lead to confusion in the clinical literature regarding the names of the organisms or the diseases they cause among clinicians who do not closely follow taxonomic changes but are still responsible for navigating the medical publications to find clinically useful information regarding invasive mycoses and their etiologic agents in order to optimize patient care [17]. In addition, there is no single source that can be used to stay abreast of changes in fungal taxonomy and literature, as descriptions of new species or revised classifications are published in various scientific journals [58], many of which lack clinical scope. Websites that serve as useful online repositories include Mycobank (http://www.mycobank.org) and Index Fungorum (http://www.indexfungorum.org). Other useful resources include the Westerdijk Fungal Biodiversity Institute (http://www.westerdijkinstitute.nl/), the Atlas of Clinical Fungi, (http://www.clinicalfungi.org/), The Yeasts website (http://theyeasts.org/) and the International Commission of *Penicillium* and *Aspergillus* (https://www.aspergilluspenicillium.org/).

## 4. Recommendations for Nomenclature Changes in Medical Mycology

The relevance of nomenclature changes to medical mycology is often unknown at first and only later once cryptic or sibling species have been further evaluated in in vitro studies, animal models, or with the publications of case reports, does the clinical significance, or lack thereof, become better understood [17,19]. Because of this and the confusion that may be present in the literature due to differences in fungal nomenclature used between the clinical and purely mycologic literature, the International Society for Human and Animal Mycology Working Group on Nomenclature of Medical Fungi has made recommendations on the adoption of new fungal names. In general, this group has proposed that the clinical arena be allowed to follow and adopt changes in nomenclature at a slower pace [17,19]. At the genus level and higher, the taxa with similar medical attributes/characteristics would be maintained and changes should be made once validated and a consensus is reached regarding new classifications and nomenclature. Taxa should not be too large as this could possibly conceal phenotypic differences of clinical importance. Conversely, taxa should not be too small as this would reduce the distinction between genus and species; however, monotypic genera do exist (e.g., *Epidermophyton* and *Lophophyton*) [19,59].

At the species level, the term species complex should be used to cover the name used in medical practice for a group of similar organisms when there is a lack of evidence of the clinical relevance of cryptic species. Once the significance of the cryptic species becomes known to the medical community, the new name can be adopted and used by clinical laboratories and medical mycologists.

## 5. Species Complexes

In the mycology literature there has been an increased use of the term species complex. However, there is no clear taxonomic definition/statute for this term and various authors have used it in different contexts [60]. Some have used it to describe a selected group of organisms that are difficult to differentiate between based on standard diagnostic means, including classic morphologic and other phenotypic characteristics and in some cases DNA barcode analysis using single targets [60]. An example of this is the *Aspergillus viridinutans* species complex within *Aspergillus* section *Fumigati*, which includes 10 closely related species, including the human and animal pathogens *A. udagawae, A. felis, A. pseudofelis, A. parafelis, A. pseudoviridinutans and A. wyomingensis* [61].

In contrast, others have used the term species complex as a substitute for the subgenus term section. Examples of this use can be found within the *Fusarium*, *Aspergillus and Trichoderma* genera [62,63]. Still others have used species complexes to group together well-described species for which there are no known or insignificant differences in clinical parameters. An example of this is the *Aspergillus niger* species complex, in which there is a lack in differences in antifungal susceptibility profiles between the various species [64]. Other examples of the use of this term in this fashion may include the *Coccidioides immitis* species complex [65,66], which is now recognized to consist of the separate species *C. immitis* and *C. posadasii* [67], the *Candida albicans* species complex, which known to consist of *C. albicans*, *C. africana* and *C. stellatoidea* [66,68,69,70,71] and the *Candida glabrata* species complex, consisting of *C. glabrata*, *C. nivariensis* and *C. bracarensis* [66,71,72,73,74]. Although *C. immitis* and *C. posadasii* may differ in their geographic distributions [75,76], no clinically relevant differences appear to exist between these two species. Interestingly, the term species complex has been used in the literature for the examples listed above, even when it is known that these consist of distinct species [60,66,71,74].

Lastly, species complex has also been employed to group together species when the taxonomy is unsettled or under debate in the literature. This has been proposed for seven separate *Cryptococcus* species (*Cryptococcus neoformans* species complex) [23], although this is still under debate and different groups have different opinions as to the lumping or splitting of these species [66,77]. Although there may be important phenotypic differences among the species, the clinical relevance of these is not fully understood. Another example where key phenotypic differences may exist but the clinical relevance is not fully known is the *Candida parapsilosis* species complex (*C. parapsilosis*, *C. metapsilosis* and *C. orthopsilosis*). The original species, *C. parapsilosis*, is known to have reduced in vitro susceptibility to the echinocandins [78], although patients often respond well to therapy [79,80,81]. It is now known that *C. metapsilosis* and *C. orthopsilosis* are hybrids and this may be of clinical relevance [82,83].

One way that clinical laboratories can use species complexes is in the reporting of preliminary microbiologic test results. If a preliminary identification of an isolate can be reported to a clinician at the species complex level, this information may be useful in making treatment decisions while further studies are performed to identify the exact species. Once the species is known, the final results should then be provided. However, clinicians should also be made aware that all species within a particular complex may not have the same antifungal susceptibility profiles. Thus, a full species identification should be provided if available. This information will then be available for clinicians, epidemiologists and other mycologists for further study.

## 6. Clinically Relevant Changes in Fungal Nomenclature and Current Controversies

Acute invasive aspergillosis and chronic pulmonary aspergillosis are primarily caused by the species *A. fumigatus, A. flavus, A. nidulans, A. niger and A. terreus* [84,85,86]. However, surveillance studies that have used molecular means of species identification have reported higher rates of cryptic species than previously appreciated. In the TRANSNET study, which included solid organ and hematopoietic stem cell transplant recipients in U.S. centers, 11% of the 218 *Aspergillus* species isolated were found to be cryptic species, including *A. lentulus* (1.8%) and *A. udagawae* (1.4%) from section *Fumigati*, *A. tubingensis* (2.8%) from section *Nigri* and *A. calidoustus* (2.8%) from section *Usti* [87]. Similarly, in the FILPOP study, a population-based survey study conducted in Spain, 14.5% of the *Aspergillus* isolates were considered to be cryptic species [86]. This may be of clinical importance as several cryptic species have reduced susceptibility to the azoles or multiple classes of clinically available antifungals. For example, section *Fumigati*, there are currently at least 63 phylogenetically distinct species, of which at least 19 have been reported to cause disease in humans and animals [88,89,90]. This includes several that were previously known as *Neosartorya* species, including *A. fischeri* (formerly *N. fischeri*), *A. hiratsukae* (formerly *N. hiratsukae*), *A. thermomutatus* (formerly *N. pseudofischeri*) and *A. udagawae* (formerly *N. udagawae*) [91,92,93,94,95]. The previously discussed *A. viridinutans* species complex also falls into section *Fumigati*. Although the importance of distinguishing between members of this complex in the clinical setting is unknown, it is important to know that the species causing infection falls within this complex as these species are often associated with chronic infections as well as reduced antifungal susceptibility and thus may be refractory to therapy [61].

Another group of clinically important fungi that has undergone major taxonomic and nomenclature changes over the last decade is that of *Scedosporium.* Previously, *Pseudallescheria boydii* and *Scedosporium apiospermum* were considered to be the same species and were identified by morphology in clinical microbiology laboratories as *P. boydii* (teleomorph) or *S. apiospermum* (anamorph) based on their ability to develop sexual structures on routine culture media. This changed when it was determined that *P. boydii* (anamorph *Scedosporium boydii*) and *Pseudallescheria apiosperma* (anamorph *S. apiospermum)* were separate species based on phylogenetic analysis [96,97]. Subsequently, other species that are morphologically identical but genetically different have been discovered through the use of molecular phylogenetics [55,96,98]. The *Scedosporium apiospermum* species complex is composed of *S. apiospermum*, *S. boydii* and *Pseudallescheria angusta* [60]. However, other *Scedosporium* species, including *S. aurantiacum, S. dehoogii and S. minutisporum*, have not been placed within this species complex due to clear phylogenetic differences among the species and those that comprise this group, as well as differences in antifungal susceptibility patterns [60,99]. The morphologically distinct species previously known as *Scedosporium prolificans* has been renamed *Lomentospora prolificans* based on significant phylogenetic differences [55]. As *L. prolificans* is highly resistant to multiple antifungals [99,100,101,102,103,104] and infections caused by this organism are extremely difficult to treat [100], the distinction between this species and those in the genus *Scedosporium* species is clinically relevant.

Recently, a revision to the taxonomy of *Cryptococcus* species that frequently cause disease in humans was proposed. In a study that included 115 isolates, *Cryptococcus neoformans* var. *grubii* and *Cryptococcus neoformans* var. *neoformans* were split into the separate species *Cryptococcus neoformans* and *Cryptococcus deneoformans*, respectively [23], while *Cryptococcus gattii* was proposed to be split into 5 distinct species (Table 3). This was based on the results from multi-locus sequence typing (MLST) based phylogenetic analysis using 11 different loci, differences in phenotypic characteristics and other means. Phenotypic characteristics that were evaluated in this study and others have included temperature, melanin content, virulence in a *Drosophila melanogaster* model, sensitivity to mycophenolic acid and growth on L-canavanine glycine bromothymol blue (CGB) agar and creatinine dextrose bromothymol blue thymine (CDBT) agar [23,66]. The authors also evaluated MALDI-TOF MS and reported that this technology could also readily distinguish between the different *Cryptococcus* species.

This proposal to divide the *Cryptococcus neoformans/gattii* species complex into different species has not been without criticism. In an editorial, Kwon-Chung et al. argued that the proposed division was premature as an insufficient number of isolates were used to make this taxonomic change [77]. A previous, larger analysis including over 2000 isolates, had showed greater genetic diversity and the possibility of even more species [77]. In addition, since loci from only 6 of the 14 chromosomes in *Cryptococcus* were used in the MLST-based phylogenetic analysis, the true extent of diversity and recombination events remains unknown. It was also argued that the proposed division is impractical for routine use in clinical microbiology laboratories. Eleven concatenated loci were used in the phylogenetic analysis that supported separating the species and even the most commonly used MLST scheme of seven concatenated loci recommended by the ISHAM Genotyping Working Group of *C. neoformans* and *C. gattii* is too complicated for clinical microbiology laboratories and even reference laboratories, especially since the loci commonly used for molecular identification of fungal species (i.e., ITS and D1/D2) are not included [77,105]. In addition, the MALDI-TOF MS score threshold used was somewhat different than the usual score cutoff value for species recognition [23,77,106] and the newly proposed species are not currently available in databases cleared by regulatory agencies for use in clinical microbiology laboratories. It should be noted that other studies have reported that lower score thresholds can be used to reliably identify fungal species [107,108,109]. However, many clinical microbiology laboratories may be reluctant to use lower score thresholds without internal validation studies. Concern was also raised regarding the possible creation of confusion between the taxonomic and clinical literature. Specifically, under the proposed nomenclature the Vancouver *C. gattii* epidemic reference strain R265 would no longer be *C. gattii* but instead would be reclassified as *C. deuterogattii*. Although it was recognized that the designation of seven separate species would be an important step for the formal recognition of the biodiversity of pathogenic *Cryptococcus* species, Kwon-Chung et al. instead proposed the use of *Cryptococcus neoformans* species complex and *Cryptococcus gattii* species complex based on these issues and our current insufficient understanding of the clinical differences among the various proposed *Cryptococcus* species. In a rebuttal, Hagen et al. defended the nomenclature changes and noted that the main advantage will be the advancement of the field through stimulation of further studies to assess for similarities and differences between the recognized species [66]. Additional work has subsequently reported that the newly proposed *Cryptococcus* species may indeed have clinically significant differences [23,66,110,111]. However, many clinical microbiology laboratories may not be able to adapt to the new nomenclature into their routine workflow in the near future, as the new species are not yet incorporated into commercially available assays and databases cleared for clinical use by regulatory agencies for diagnosis or species identification.

*Fusarium* species are significant causes of invasive infections in highly immunocompromised hosts [112,113]. In addition, infections including keratitis and onychomycosis, can also occur in immunocompetent patients [27,114]. Human infections can be caused by species grouped within 8 different species complexes, including: *Fusarium solani* species complex, *Fusarium oxysporum* species complex, *Fusarium fujikuroi* species complex, *Fusarium chlamydosporum* species complex, *Fusarium dimerum* species complex, *Fusarium incarnatum-equiseti* species complex, *Fusarium sambucinum* species complex and *Fusarium tricinctum* species complex [25], although most infections are caused by members of the *F. solani* and *F. oxysporum* species complexes [112]. The *F. solani* species complex encompass at least 60 phylogenetically distinct species and in addition to causing disease in humans and animals, also includes a number of important agricultural pathogens [115]. Traditionally, clinical microbiology laboratories have identified and reported these isolates as *F. solani* species complex and some reference laboratories also report the specific haplotype based on MLST of the translation elongation factor 1α and RNA polymerase II gene. Although some members of this complex have received formal species names (e.g., *F. petroliphilum* [halplotype 1], *F. keratoplasticum* [haplotype 2], *F. falciforme* [haplotype 3+4] and *F. solani* [haplotype 5]) many others have not [116,117].

Recently, it has been proposed that members of the *F. solani* species complex be moved to the genus *Neocosmospora* based on the results of phylogenetic analysis [53] and new species previously classified only as haplotypes have been described [118]. This includes the species *N. petroliphila* (*F. petroliphilum*), *N. keratoplastica* (*F. keratoplasticum*), *N. falciformis* (*F. falciforme*) and *N. solani* (*F. solani*), along with the new species *N. gamsii* (haplotype 7), *N. suttoniana* (haplotype 20) and *N. catenata* (haplotype 43). Others have argued against renaming members of the *F. solani* species complex based on the long-standing, historical concept of this genus [119]. In order to provide up-to-date information to both clinicians and clinical microbiologists, as well as facilitate their ability to find relevant information in the medical literature, the reports generated by our reference mycology laboratory provide both the name commonly used in the medical literature as well as the new nomenclature. For example, for a recent species identification of an isolate cultured from the cornea of a patient, the name frequently found in the clinical literature, *Fusarium falciforme*, was provided along with a statement that the species is now known as *Neocosmospora falciformis*.

## 7. Summary

The field of medical mycology is rapidly changing due to the introduction of new molecular and proteomic technologies. New species are rapidly being discovered in the environment, as are new etiologic agents of disease in humans and animals. The adoption of these technologies, along with the abandonment of the dual nomenclature system, has led to marked changes in fungal taxonomy and nomenclature, as organisms previously thought to be unrelated are now recognized as being genetically similar. Conversely, we are now learning that species that were previously considered to be related are in fact very different from each other. The rapidity of these changes has caused concern among some medical mycologists and clinicians that the nomenclature changes may lead to negative clinical consequences, as the ever-changing literature could cause confusion among those who are responsible direct patient care. To mitigate this possibility, it has been proposed that medical mycology, specifically clinicians and clinical microbiology laboratories, may need to adopt changes in fungal nomenclature and taxonomy at a more measured pace. In addition, clinical microbiology and reference laboratories should provide useful information that will aide clinicians in this endeavor. This includes keeping abreast of changes in taxonomy and nomenclature, serving as a resource for clinicians as to previous names that may be published in the literature, as well as the clinical significance of the new classifications. An example of how the clinical laboratories may provide up-to-date information as well as species names that are prevalent in the clinical literature is provided above (*Neocosmospora falciformis* and *Fusarium falciforme*). The discovery of new fungal species capable of causing disease in humans and animals and the reclassification of various groups will continue as our knowledge of fungal diversity increases. However, this should not impede clinicians in their treatment of patients with fungal infections.

## Figures and Tables

**Table 1:** Examples of recently described and medically relevant fungi.

Species	Family	Order	Sites & Infections in Humans	Reference
*Apophysomyces mexicanus*	Saksenaeaceae	Mucorales	Necrotizing fasciitis	[31]
*Aspergillus citrinoterreus*	Aspergillaceae	Eurotiales	Pulmonary infection	[32]
*Aspergillus suttoniae*	Aspergillaceae	Eurotiales	Human sputum	[33]
*Aspergillus tanneri*	Aspergillaceae	Eurotiales	Lung, gastric abscess	[34]
*Candida auris*	Incertae sedis	Saccharomycetales	Various sites, candidemia	[35]
*Curvularia americana*	Pleosporaceae	Pleosporales	Nasal sinus, bone marrow	[36]
*Curvularia chlamydospora*	Pleosporaceae	Pleosporales	Nasal sinus, nail	[36]
*Emergomyces canadensis*	Ajellomycetaceae	Onygenales	Pneumonia, fungemia	[37,38]
*Exophiala polymorpha*	Herpotrichiellaceae	Chaetothyriales	Subcutaneous & cutaneous infections	[39]
*Paracoccidioides lutzi*	Ajellomycetaceae	Onygenales	Various	[40]
*Rasamsonia aegroticola*	Aspergillaceae	Eurotiales	Pulmonary infections	[41,42]
*Spiromastigoides albida*	Spiromastigaceae	Onygenales	Lung biopsy	[43]

**Table 2:** Examples of fungal nomenclature changes in medically relevant fungi.

New Name	Previous Name	Family	Order	Reference
**Yeasts**				
*Apiotrichum mycotoxinivorans*	*Trichosporon mycotoxinivorans*	Trichosporonaceae	Trichosporonales	[44]
*Candida duobushaemulonii*	*Candida haemulonii* group II	Incertae sedis	Saccharomycetales	[45]
*Kluyveromyces marxianus*	*Candida kefyr*	Saccharomycetaceae	Saccharomycetales	[46]
*Magnusiomyces capitatus*	*Blastoschizomyces capitatus/* *Geotrichum capitatum*	Dipodascaceae	Saccharomycetales	[47]
*Meyerozyma guilliermondii*	*Candida guilliermondii*	Debaryomycetaceae	Saccharomycetales	[48]
**Moulds**				
*Blastomyces helicus*	*Emmonsia helica*	Onygenaceae	Onygenalses	[33]
*Blastomyces parvus*	*Emmonsia parva*	Onygenaceae	Onygenales	[33]
*Curvularia australiensis*	*Bipolaris australiensis*	Pleosporaceae	Pleosporales	[49]
*Cuvularia hawaiiensis*	*Bipolaris hawaiiensis*	Pleosporaceae	Pleosporales	[49]
*Curvularia spicifera*	*Bipolaris spicifera*	Pleosporaceae	Pleosporales	[49]
*Lichtheimia corymbifera*	*Absidia corymbifera*	Lichtheimiaceae	Mucorales	[50]
*Neocosmospora solani*	*Fusarium solani*	Nectriaceae	Hypocreales	[51]
*Purpureocillium lilacinum*	*Paecilomyces lilacinus*	Ophiocrodycipitaceae	Hypocreales	[52]
*Aspergillus thermomutatus*	*Neosartorya pseudofischeri*	Aspergillaceae	Eurotiales	[53,54]
*Aspergillus udagawae*	*Neosartorya udagawae*	Aspergillaceae	Eurotiales	[54]
*Rasamsonia argillacea*	*Geosmithia argilacea*	Aspergillaceae	Eurotiales	[37]
*Scedosporium boydii*	*Pseudallescheria boydii*	Microascaceae	Microascales	[55]
*Verruconis gallopava*	*Ochroconis gallopava*	Sympoventuriaceae	Venturiales	[56]
*Talaromyces marneffei*	*Penicillium marneffei*	Aspergillaceae	Eurotiales	[57]

**Table 3:** Proposed names for *Cryptococcus neoformans* and *C. gattii* species [23].

Current Name	Molecular Type	Proposed Name
*Cryptococcus neoformans* var. *grubii*	VNI, VNII, VNB	*Cryptococcus neoformans*
*Cryptococcus neoformans* var. *neoformans*	VNIV	*Cryptococcus deneoformans*
*Cryptococcus gattii*	VGI	*Cryptococcus gattii*
	VGIII	*Cryptococcus bacillisporus*
	VGII	*Cryptococcus deuterogattii*
	VGIV	*Cryptococcus tetragattii*
	VGIV/VGIIIc	*Cryptococcus decagattii*
Serotypes AD hybrid	VNIII	*Cryptococcus neoformans* x *Cryptococcus deneoformans* hybrid
Serotypes DB hybrid	AFLP8	*Cryptococcus deneoformans* x *Cryptococcus gattii* hybrid
Serotypes AB hybrid	AFLP9	*Cryptococcus neoformans* x *Cryptococcus gattii* hybrid
Serotypes AB hybrid	AFLP11	*Cryptococcus neoformans* x *Cryptococcus deuterogattii* hybrid
